# Cortactin regulates endo-lysosomal sorting of AMPARs via direct interaction with GluA2 subunit

**DOI:** 10.1038/s41598-018-22542-z

**Published:** 2018-03-07

**Authors:** Gabrielle T. Parkinson, Sophie E. L. Chamberlain, Nadia Jaafari, Matthew Turvey, Jack R. Mellor, Jonathan G. Hanley

**Affiliations:** 1 Centre for Synaptic Plasticity and School of Biochemistry, Biomedical Sciences Building, University of Bristol, University Walk, Bristol, BS8 1TD UK; 2 Centre for Synaptic Plasticity and School of Physiology, Pharmacology & Neuroscience, Biomedical Sciences Building, University of Bristol, University Walk, Bristol, BS8 1TD UK

## Abstract

AMPA receptor (AMPAR) trafficking is a key determinant of synaptic strength and synaptic plasticity. Under basal conditions, constitutive trafficking maintains surface AMPARs by internalization into the endosomal system, where the majority are sorted and targeted for recycling back to the plasma membrane. NMDA receptor (NMDAR)-dependent Long-Term Depression (LTD) is characterised by a reduction in synaptic strength, and involves endosomal sorting of AMPARs away from recycling pathways to lysosomes. The mechanisms that determine whether AMPARs are trafficked to lysosomes or to recycling endosomes, especially in response to NMDAR stimulation, are unclear. Here, we define a role for the actin-regulatory protein cortactin as a mediator of AMPAR endosomal sorting by direct interaction with the GluA2 subunit. Disrupting GluA2-cortactin binding in neurons causes the targeting of GluA2/A3-containing receptors to lysosomes and their consequent degradation, resulting in a loss of surface and synaptic GluA2 under basal conditions and an occlusion of subsequent LTD expression. Furthermore, we show that NMDAR stimulation causes a dissociation of endogenous cortactin from GluA2 via tyrosine phosphorylation of cortactin. These results demonstrate that cortactin maintains GluA2/A3 levels by directing receptors away from lysosomes, and that disrupting GluA2-cortactin interactions to target GluA2/A3 to lysosomes is an essential component of LTD expression.

## Introduction

Long-term synaptic plasticity is thought to underlie learning and memory and the fine-tuning of neural circuitry during development. AMPA receptors (AMPARs) mediate the majority of fast excitatory synaptic transmission in the brain, and plasticity at excitatory synapses requires alterations in AMPAR number at the synaptic plasma membrane brought about by regulated trafficking of AMPAR-containing vesicles. A decrease in synaptic strength involves a removal of AMPARs from synapses in Long-Term Depression (LTD), whereas an increase in the number of synaptic AMPARs leads to increased synaptic strength during Long-Term Potentiation (LTP)^1–4^. In addition, a number of neurological disorders such as ischaemia, traumatic brain injury and Alzheimer’s involve aberrant AMPAR trafficking, which can lead to synaptic dysfunction and neuronal cell death^5–8^.

AMPARs undergo constitutive trafficking involving endocytosis, endosomal sorting and recycling to the plasma membrane. NMDA receptor (NMDAR)-dependent LTD requires not only an increase in clathrin-mediated endocytosis to internalize AMPARs from the plasma membrane, but also endosomal sorting steps whereby internalized AMPARs are targeted for lysosomal degradation, instead of recycling to the plasma membrane^9–12^. The specific AMPAR-associated mechanisms that regulate the switch from endosomal recycling to lysosomal degradation in response to LTD induction are incompletely understood, and defining these mechanisms is therefore critically important to our understanding of learning and memory processes, and of relevant neurological disorders. It has been suggested previously that NMDAR-dependent lysosomal targeting and degradation of AMPARs is regulated by the GluA2 subunit and its associated protein interactions^9^, but the identity of the interacting proteins and the details of the regulatory mechanisms are unknown.

The actin cytoskeleton is central to the regulation of intracellular membrane trafficking by exerting mechanical forces that contribute to the changes in membrane geometry required for vesicle biogenesis and formation of tubular domains on endosomes^13^. The Arp2/3 complex is the major catalyst for the formation of branched actin networks that mediate such membrane dynamics, and Arp2/3 regulators such as the WASH complex play a critical role in the sorting of cargo into the recycling pathway from early endosomes^12,14^. Cortactin enhances Arp2/3-mediated actin polymerization, and is also thought to stabilize preexisting actin filaments^15,16^. In non-neuronal cells, cortactin is recruited to endocytic sites, suggesting a role in regulating actin polymerization during clathrin-mediated endocytosis^17^, and it is also localized to subdomains of sorting endosomes, suggesting it plays a role in recycling of specific cargo^18^. In neurons, cortactin regulates dendritic spine morphology via its ability to bind to, and presumably regulate the stability and/or polymerization of actin filaments^19^. Importantly, cortactin was previously identified in a proteomics screen for endogenous AMPAR associated proteins^20^, however the interaction has not been further studied, and its role in trafficking in neurons is unknown.

Here, we show that cortactin binds directly to a membrane-proximal region of GluA2, and this interaction is required for GluA2-dependent AMPAR recycling under basal conditions. Mutant cortactin that does not bind GluA2 causes lysosomal targeting and degradation of GluA2/A3-containing AMPARs, and a consequent reduction in surface and synaptic GluA2, which occludes subsequent induction of hippocampal LTD.

## Results

### Cortactin binds a membrane-proximal region of GluA2

**Figure 1:**
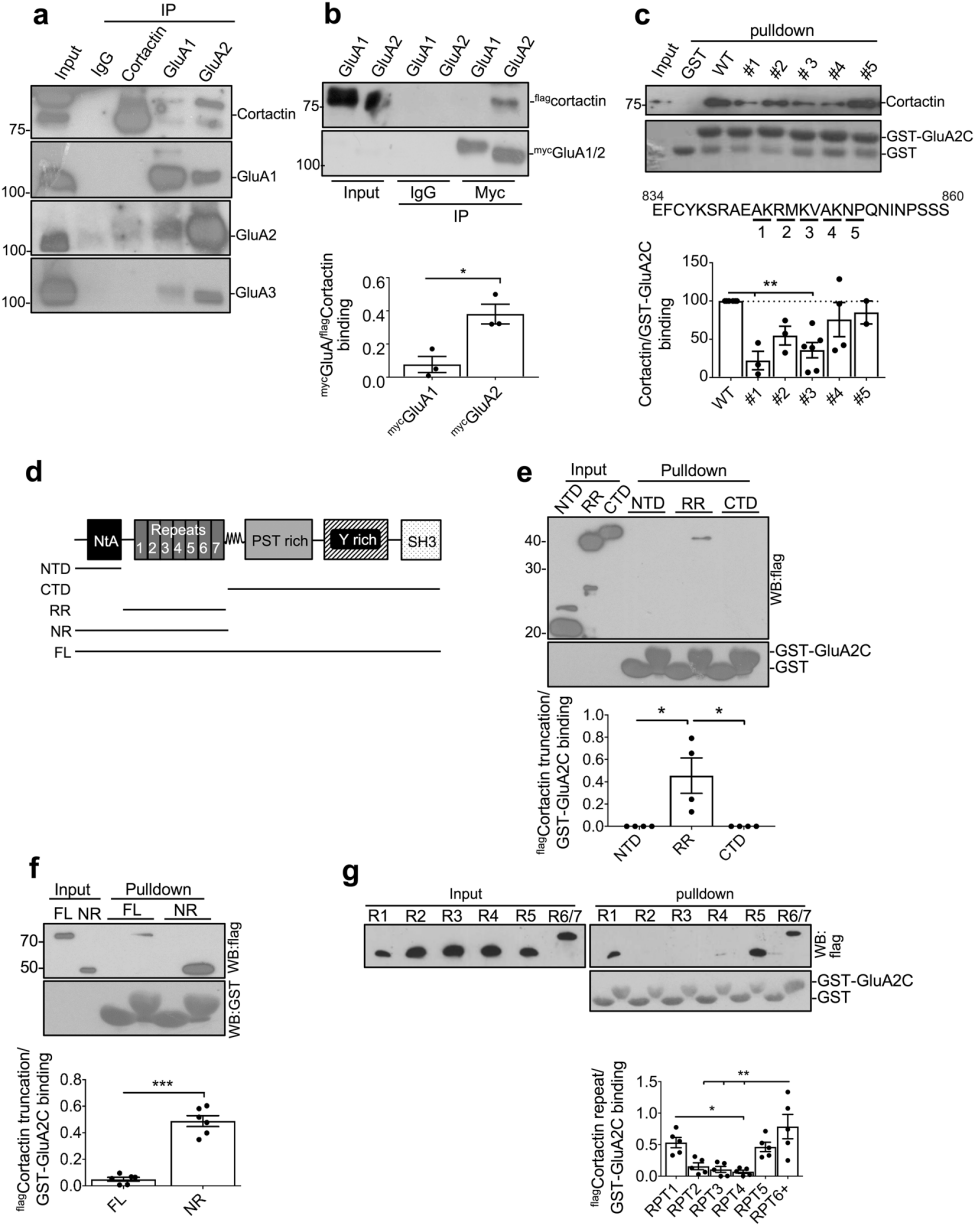
Cortactin repeat region binds a membrane-proximal region of GluA2. (**a**) Endogenous cortactin and GluA2 associate in neurons. Lysates of cortical neurons were immunoprecipitated with cortactin, GluA1 or GluA2 antibody or control IgG. Proteins were detected by Western blotting. Input is 1% of offered protein. (**b**) Cortactin interacts with GluA2, but not GluA1 in heterologous cells. Lysates of HEK293 cells transfected with ^flag^cortactin, ^myc^GluA2, or ^myc^GluA1 were immunoprecipitated with Myc tag antibody or control IgG. Proteins were detected by Western blotting. Input is 1% of offered protein. Graph shows quantification of cortactin binding to Myc-GluA2, values represent mean ± SEM. Unpaired two-tailed student’s t-test (t(4) = 3.963, *p < 0.05, n = 3). (**c**) Cortactin binds directly to a membrane-proximal region of GluA2. GST, GST-GluA2 CT, or GST-GluA2 CT mutants as shown were immobilized on glutathione-agarose and incubated with purified his_6_-cortactin. Proteins were detected by Western blotting. Input is 0.2% of offered protein. Graph shows quantification of cortactin binding to GST-GluA2 CT, values represent mean ± SEM. One-way ANOVA with Dunnett’s test for multiple comparisons (F(5,18) = 4.402, **p < 0.01, n = 3–6). Sequence shows the membrane-proximal region of GluA2 C-terminal tail, illustrating the residues mutated to alanine in the constructs used for the pulldown. (**d**) Schematic illustrating the truncations used in E-G, below. (**e**) GluA2 binds directly to the repeat region of cortactin. GST or GST-GluA2 CT were immobilized on glutathione-agarose and incubated with purified his_6_-^flag^cortactin truncations as shown. Proteins were detected by Western blotting. Input is 0.2% of offered protein. Graph shows quantification of cortactin binding to GST-GluA2 CT, values represent mean ± SEM. One-way ANOVA with Bonferroni’s test for multiple comparisons (F(2,9) = 8.192, **p < 0.01, n = 4). (**f**) C-terminal region of cortactin inhibits the interaction with GluA2. GST or GST-GluA2 CT were immobilized on glutathione-agarose and incubated with purified his_6_-^flag^cortactin full-length or NR truncation as shown. Proteins were detected by Western blotting. Input is 0.2% of offered protein. Graph shows quantification of cortactin binding to GST-GluA2 CT, values represent mean ± SEM. Unpaired two-tailed student’s t-test (t(10) = 10.07, ***p < 0.0001, n = 6). (**g**) Cortactin repeats 1, 5 and 6 bind GluA2. GST-GluA2 immobilized on glutathione-agarose was incubated with purified his_6_-^flag^cortactin isolated repeats 1–6. Proteins were detected by Western blotting. Input is 0.2% of offered protein. Graph shows quantification of cortactin binding to GST-GluA2 CT, values represent mean ± SEM. One-way ANOVA with Bonferroni’s test for multiple comparisons (F(5,24) = 8.946, **p < 0.01, n = 5).

Cortactin was previously identified as associating with AMPARs in endogenous protein complexes^20^. To confirm this, we carried out co-immunoprecipitations (co-IPs) from neuronal lysates (Fig. 1a). Cortactin was robustly co-IP’ed with GluA2, and only weakly with GluA1, suggesting that cortactin preferentially associated with GluA2-containing AMPARs in neurons. Cortactin-GluA2 complexes were weakly detectable in cortactin immunopellets, while cortactin-GluA1 and cortactin-GluA3 complexes were undetectable (Fig. 1a). To explore the subunit-selectivity of the interaction further, we carried out co-IPs from HEK293 cells transfected with flag-tagged cortactin and Myc-tagged GluA1 or GluA2. Cortactin showed a markedly stronger interaction with GluA2 compared to GluA1 (Fig. 1b). To investigate whether cortactin binds directly to AMPAR subunits, we performed GST-pulldown assays with GluA2 C-terminal tail and purified his_6_-cortactin. Figure 1c shows that cortactin bound robustly and selectively to GST-GluA2. We also explored the region of GluA2 C-terminus that binds cortactin by performing pulldowns with GST-GluA2 containing pairwise alanine substitutions, and found that a region close to the transmembrane domain was critical for cortactin binding. Interestingly, we found that the same mutations shown previously to disrupt the interaction with NSF^21^ also reduced binding to cortactin (Fig. 1c). Cortactin is a multi-domain protein, comprising an N-terminal acidic region (NTA), which includes the Arp2/3 binding site, six tandem repeats, of which repeat 4 is required for binding to F-actin, an α-helical domain, a proline-rich region and an SH3 domain^22^. To investigate the region of cortactin involved in binding GluA2, we carried out pulldowns with GST-GluA2 and truncation mutants of cortactin. GluA2 showed a robust interaction with the repeat region (amino acids 85–336; Fig. 1d,e), but not the N-terminal NTA domain (amino acids 1–84) nor the C-terminal region (amino acids 337–546). Moreover, a truncation comprising the NTA and repeat region (NR) bound GluA2 markedly stronger than the full-length protein, suggesting an inhibitory influence of the C-terminal part of the protein (Fig. 1d,f). These results demonstrated an interaction between GluA2 and the repeat region of cortactin, so we asked whether the isolated repeats bind GluA2, and analyzed the binding of GST-GluA2 to isolated his_6_-tagged repeats. GST-GluA2 interacted robustly with purified repeat regions 1,5 and 6, but only weakly with 2, 3 and 4 (Fig. 1g).

### GluA2 binds repeats 1, 5 and 6 of cortactin

**Figure 2:**
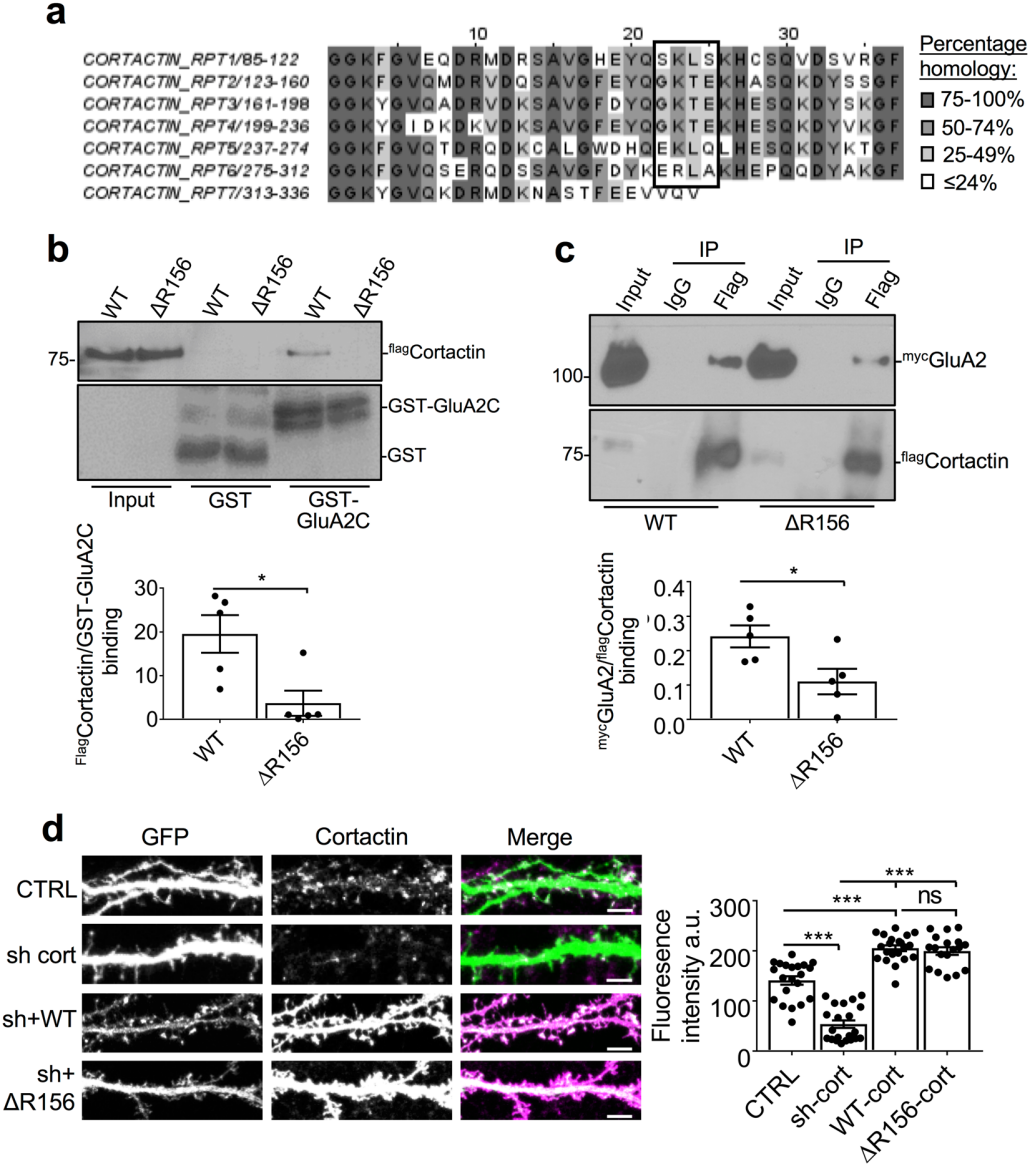
Mutations in cortactin repeats 1, 5 and 6 block GluA2 binding. (**a**) Schematic showing sequence alignment of cortactin repeats. Boxed region shows the sequence where 1, 5 and 6 are distinct from 2, 3 and 4. (**b**) ΔR156 mutations disrupt cortactin binding to GST-GluA2 CT. GST or GST-GluA2 CT were immobilized on glutathione-agarose and incubated with purified his_6_-WT cortactin or his_6_-ΔR156 cortactin. Proteins were detected by Western blotting. Input is 0.2% of offered protein. Graph shows quantification of cortactin binding to GST-GluA2 CT, values represent mean ± SEM. Unpaired two-tailed student’s t-test (t(8) = 3.053, *p < 0.05, n = 5). (**c**) ΔR156 mutations disrupt cortactin binding to full-length GluA2. Lysates of HEK293 cells transfected with ^flag^WT-cortactin or ^flag^ΔR156-cortactin and ^myc^GluA2 were immunoprecipitated with Flag tag antibody or control IgG. Proteins were detected by Western blotting. Input is 1% of offered protein. Graph shows quantification of cortactin binding to ^myc^GluA2, values represent mean ± SEM. Unpaired two-tailed student’s t-test (t(8) = 2.682, *p < 0.05, n = 5). (**d**) Characterization of molecular replacement constructs. Hippocampal neurons were transfected with the constructs depicted in the schematic, and stained with cortactin antibody (magenta). Green channel shows GFP fluorescence. Representative 45 μm lengths of dendrites are shown, scale bar = 5 µm. Graph shows fluorescence intensity for cortactin staining, values represent mean ± SEM. One-way ANOVA with Bonferroni’s test for multiple comparisons (F(3,77) = 0.9918, ***p < 0.0001, n = 18–21).

In order to study this interaction in neurons, we wanted to generate a mutant cortactin that would not interact with GluA2. Based on our results in Fig. 1g, we reasoned that sequence differences between repeats 1,5,6 and 2,3,4 would be critical for determining GluA2 binding. We scrutinized the amino acid sequence of the 6 repeats, which show close homology^23^; (Fig. 2a), and found that repeat 2, 3 and 4 all contain GKTE where 1, 5 and 6 contain SKLS, EKLQ, and ERLA, respectively (see boxed region in Fig. 2a). Therefore, we generated a full-length cortactin mutant with GKTE substitutions in all three of the repeat regions that bind GluA2 (repeats 1, 5 and 6). This mutant, hereafter referred to as ΔR156, showed a dramatically reduced interaction with GST-GluA2 C-terminus (Fig. 2b) and the full-length GluA2 subunit (Fig. 2c) compared to wild-type cortactin. To study the role of the GluA2-cortactin interaction in neurons, we generated molecular replacement constructs that express a previously-characterized cortactin shRNA^19^ as well as GFP or shRNA-resistant GFP-tagged WT or ΔR156-cortactin. Transfecting cultured hippocampal neurons with the construct encoding cortactin shRNA and GFP resulted in cortactin expression at around 35% of control levels (Fig. 2d). Co-expression of cortactin shRNA with sh-resistant WT- or ΔR156-cortactin (molecular replacement constructs) resulted in cortactin expression levels that were approximately 45% higher than endogenous cortactin (Fig. 2d). However, we acknowledge that the immunofluorescence signal from the recombinant proteins is saturated, resulting in an underestimate of the expression level.

### Interaction with GluA2 does not affect dendritic F-actin, and is not necessary for the maintenance of spine or synapse density by cortactin

**Figure 3:**
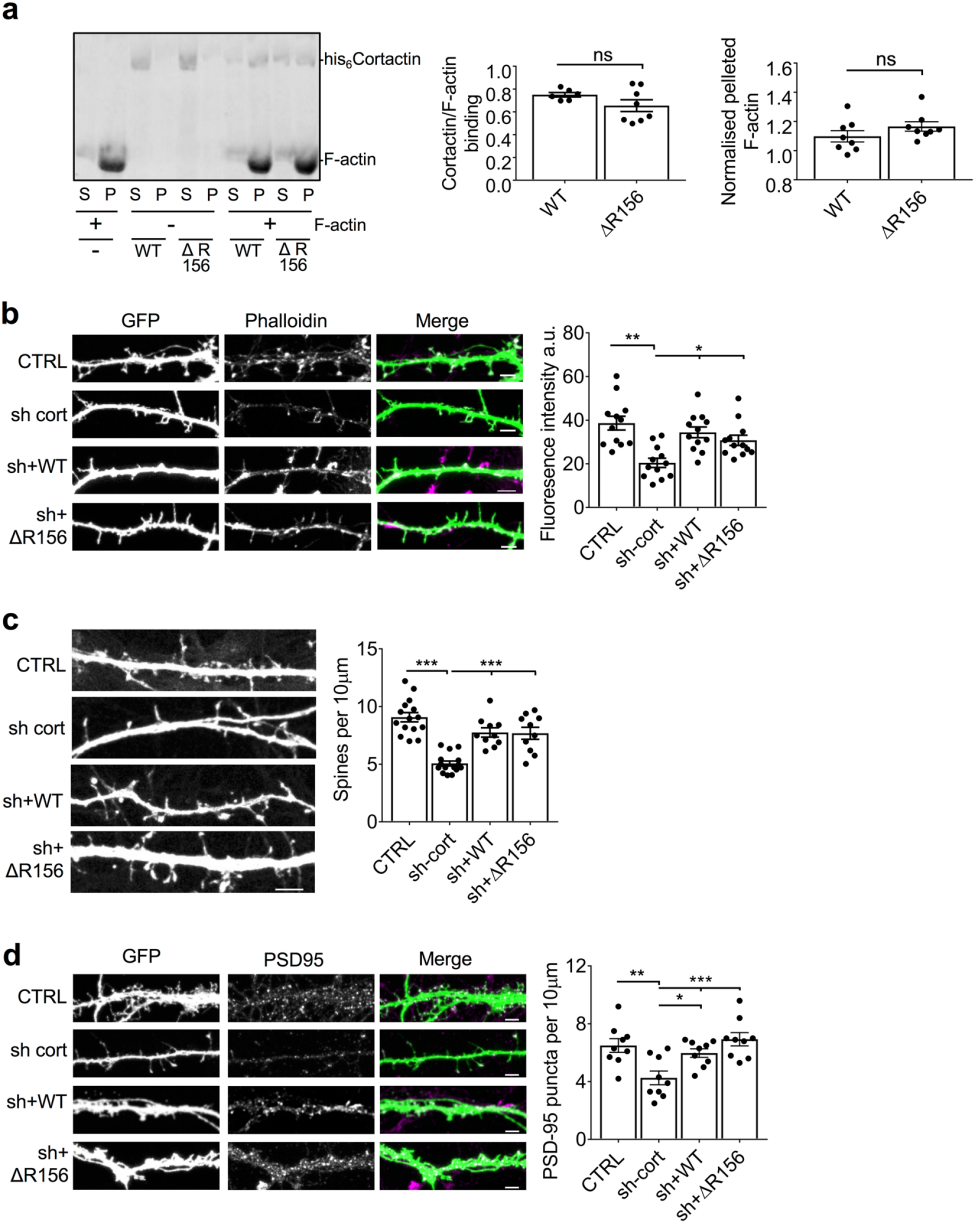
Cortactin ΔR156 mutations have no effect on binding to F-actin *in vitro*, no effect on dendritic F-actin or on spine density. (**a**) ΔR156 mutations do not affect cortactin binding to F-actin. Purified and clarified his_6_ WT-cortactin, and his_6_ ΔR156-cortactin were incubated with or without 5 μm F-actin and subjected to ultracentrifugation to pellet actin filaments and associated proteins. Protein content of the pellet (P), and supernatant (S) was analysed by Coomassie staining. Left graph shows quantification of cortactin binding to F-actin (pellet/supernatant ratio), values represent mean ± SEM. Unpaired two-tailed student’s t-test (t(12) = 1.525, n = 6–8). Right graph shows proportion of pelleted F-actin in the presence of cortactin compared to absence of cortactin, values represent mean ± SEM. Unpaired two-tailed student’s t-test (t(14) = 1.336, n = 8). (**b**) ΔR156 mutations do not affect dendritic F-actin total levels. Hippocampal neurons transfected with GFP, cortactin shRNA + GFP, cortactin shRNA + sh-resistant GFP-WT-cortactin or cortactin shRNA + sh-resistant GFP-ΔR156-cortactin were stained with AlexaFluor647-phalloidin (magenta). Representative 45 μm lengths of dendrites are shown, scale bar = 5 µm. Graphs show fluorescence intensity for phalloidin staining, values represent mean ± SEM. One-way ANOVA with Bonferroni’s test for multiple comparisons (F(3,44) = 0.8754, *p < 0.05, **p < 0.01, n = 12). (**c**) ΔR156 mutations do not affect dendritic spine density. Hippocampal neurons transfected with molecular replacement constructs as in F. Representative 45 μm lengths of dendrites are shown, scale bar = 5 µm. Graph shows the number of protrusions per 10 μm dendrite, values represent mean ± SEM. One-way ANOVA with Bonferroni’s test for multiple comparisons (F(3,38) = 2.549, ***p < 0.001, n = 10–11). (**d**) ΔR156 mutations do not affect dendritic synapse number. Hippocampal neurons transfected with GFP, cortactin shRNA + GFP, cortactin shRNA + sh-resistant GFP-WT-cortactin or cortactin shRNA + sh-resistant GFP-ΔR156-cortactin were stained with PSD-95 antibodies (magenta). Representative 45 μm lengths of dendrites are shown, scale bar = 5 µm. Graph shows the number PSD-95 puncta per 10 μm dendrite, values represent mean ± SEM. One-way ANOVA with Bonferroni’s test for multiple comparisons (F(3,32) = 0.4391, *p < 0.05, **p < 0.01, n = 9).

Since cortactin is an actin-binding protein, and the GluA2 binding mutations are close to the F-actin binding site in repeat 4, we analyzed whether the ΔR156 mutations affected F-actin binding. High-speed sedimentation assays using purified actin polymerized *in vitro* demonstrated that ΔR156-cortactin bound and stabilized F-actin to a similar level as WT-cortactin (Fig. 3a).

As a further confirmation that the ΔR156 mutation has no effect on the actin cytoskeleton, we expressed the molecular replacement constructs in cultured hippocampal neurons and analyzed F-actin by staining with AlexaFluor647-conjugated phalloidin. Cortactin knockdown by shRNA caused a significant reduction in F-actin levels, which was fully rescued by either sh-resistant WT- or ΔR156-cortactin (Fig. 3b). This demonstrated that, while cortactin is an important regulator of the actin cytoskeleton in neuronal dendrites, its interaction with GluA2 is not involved.

It has been demonstrated previously that cortactin is concentrated in dendritic spines, and targeting of the protein to spines requires the repeat region. Moreover, the same study reported that shRNA-mediated knockdown of cortactin caused a loss of dendritic spines in cultured neurons^19^. To investigate whether the interaction with GluA2 is required for maintenance of dendritic spine density by cortactin, we analyzed spine density in neurons expressing cortactin shRNA or molecular replacement constructs. Consistent with a previous report^19^, we found that cortactin shRNA caused a significant reduction in spine density (Fig. 3c). Co-expression of either sh-resistant WT-cortactin or ΔR156-cortactin rescued this effect, demonstrating that cortactin-ΔR156 expression does not affect basal dendritic spine density, and hence strongly suggesting that GluA2-cortactin interactions do not play a role in dendritic spine maintenance. We also analyzed PSD-95 as a marker for excitatory synapses. Cortactin shRNA caused a significant reduction in the density of PSD-95 positive puncta, which was fully rescued by either sh-resistant WT- or ΔR156-cortactin (Fig. 3d), demonstrating that, while cortactin is involved in maintaining excitatory synapses, the interaction with GluA2 is not required.

Taken together, these results demonstrate that the ΔR156 mutations do not affect actin polymerization, spine density or synapse density, and hence that the interaction with GluA2 is not necessary for these aspects of cortactin function.

### Disrupting GluA2-cortactin interaction causes a specific reduction in surface and synaptic levels of endogenous GluA2

**Figure 4:**
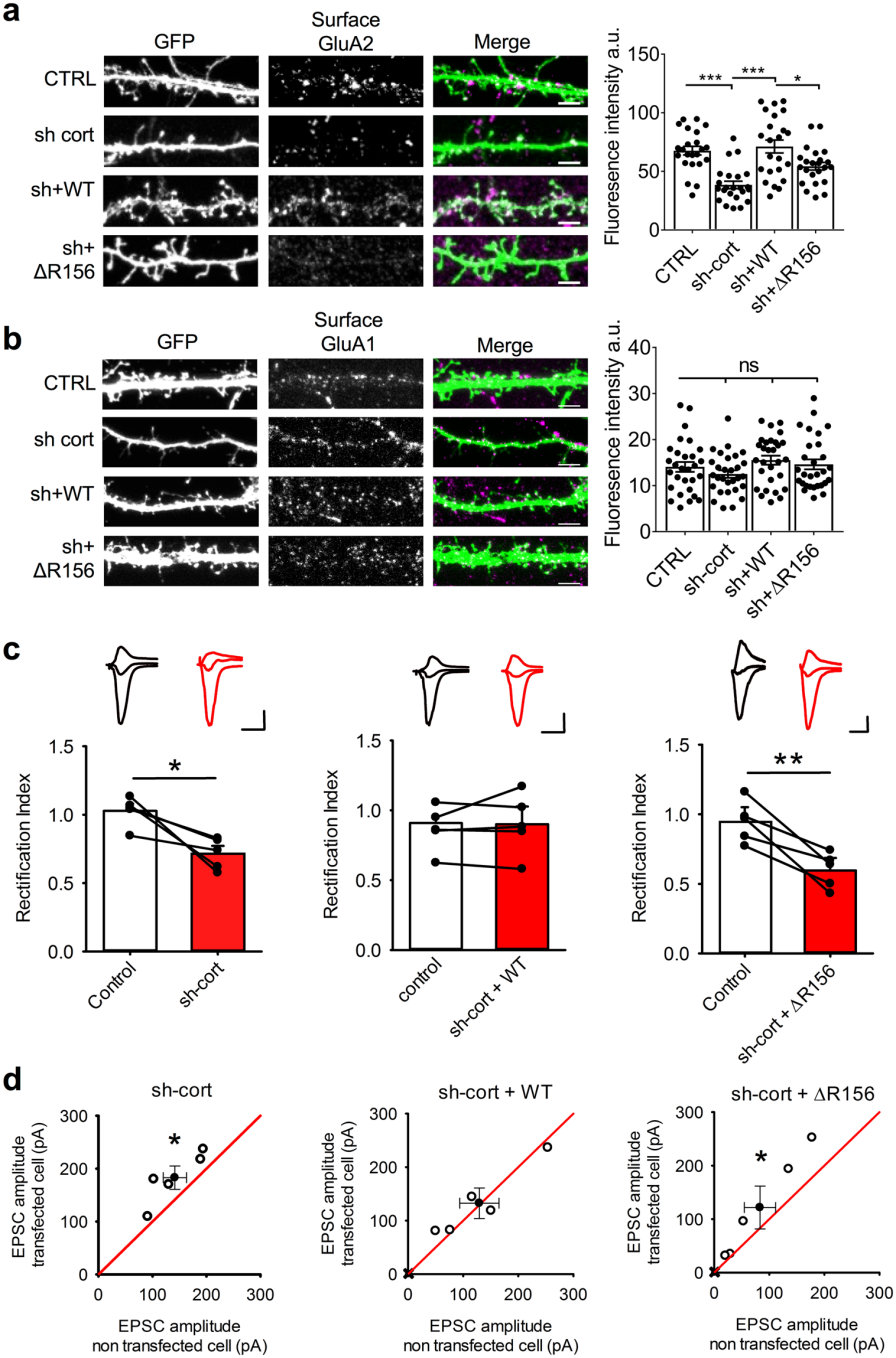
ΔR156-cortactin expression reduces surface and synaptic GluA2 under basal conditions. (**a**) ΔR156-cortactin reduces surface levels of endogenous GluA2. Live hippocampal neurons transfected with GFP, cortactin shRNA + GFP, cortactin shRNA + sh-resistant GFP-WT-cortactin or cortactin shRNA + sh-resistant GFP-ΔR156-cortactin were stained with GluA2 antibody to label surface receptors. Representative 45 μm lengths of dendrites are shown, scale bar = 5 µm. Graph shows fluorescence intensity for surface GluA2 staining, values represent mean ± SEM. One-way ANOVA with Bonferroni’s test for multiple comparisons (F(3,88) = 5.593, *p < 0.05, **p < 0.01, n = 23). (**b**) ΔR156-cortactin has no effect on surface levels of endogenous GluA1. Live hippocampal neurons transfected with constructs as in A, were stained with GluA1 antibody to label surface receptors. Representative 45 μm lengths of dendrites are shown, scale bar = 5 µm. Graph shows fluorescence intensity for surface GluA1 staining, values represent mean ± SEM. One-way ANOVA with Bonferroni’s test for multiple comparisons (F(3,112) = 0.6872, n = 29). (**c**) ΔR156-cortactin causes synaptic expression of inwardly rectifying AMPARs. Graphs show rectification indices of transfected versus neighbouring non-transfected control cells. Left, cortactin shRNA; middle, cortactin shRNA + sh-resistant GFP-WT-cortactin; right, cortactin shRNA + sh-resistant GFP-ΔR156-cortactin. Bars represent mean ± SEM, lines represent individual cells (n = 5 cells per condition). Insets show representative traces recorded at −70, 0 and +40 mV. Scale bars for all traces shown are 50 pA/50 ms. *p < 0.05, **p < 0.01, t-test. (**d**) ΔR156-cortactin causes synaptic expression of AMPARs with increased EPSC amplitudes. Graphs show EPSC amplitudes of transfected versus neighbouring non-transfected control cells. Left, cortactin shRNA; middle, cortactin shRNA + sh-resistant GFP-WT-cortactin; right, cortactin shRNA + sh-resistant GFP-ΔR156-cortactin. Open circles represent recordings of neurons from transfected and neighbouring non-transfected control cell using the same presynaptic stimulation. Filled circles represent mean ± SEM. Solid line is unity. Scale bars for all traces are 40 pA/ 50 ms. n = 5 cells per condition, *p < 0.05, t-test.

To investigate the role of cortactin in AMPAR trafficking, we initially carried out immunocytochemistry on cultured hippocampal neurons transfected with cortactin shRNA or molecular replacement constructs. Surface staining of live neurons with GluA1 or GluA2 antibodies revealed that cortactin knockdown caused a significant reduction in surface GluA2, which was fully rescued by sh-resistant WT-cortactin but not by sh-resistant ΔR156-cortactin (Fig. 4a). In contrast, surface GluA1 staining was unaffected by cortactin knockdown or ΔR156 expression (Fig. 4b). To assess the significance of the selective reduction in GluA2 on synaptic function, we analyzed AMPAR-mediated synaptic transmission using whole-cell patch-clamp electrophysiological recordings of CA1 neurons in organotypic slices. We measured AMPAR excitatory postsynaptic currents (EPSCs) at three holding potentials (−70 mV, 0 mV, and + 40 mV) and calculated the rectification index (RI) as the ratio of the slope 0 to + 40 mV and −70 to 0 mV. Hence, RI < 1 corresponds to increased inward rectification. As expected, AMPAR EPSCs in non-transfected neurons showed no detectable rectification (RI 1.03 ± 0.05, n = 5), suggesting that most synaptic AMPARs contained GluA2 subunits (Fig. 4c). Cortactin knockdown by shRNA caused a significant inward rectification (RI 0.72 ± 0.06, n = 5, *p* < 0.05), indicating that some GluA2-containing AMPARs were replaced with GluA2-lacking AMPARs at synapses (Fig. 4c). The increase in rectification was fully rescued by co-expression of sh-resistant WT-cortactin (Fig. 4c; RI control 0.91 ± 0.05 vs transfected 0.90 ± 0.13, n = 5), but not by sh-resistant ΔR156-cortactin (Fig. 4c; RI control 0.95 ± 0.11 vs transfected 0.60 ± 0.09, n = 5, *p* < 0.01). GluA2-lacking AMPARs have a higher conductance compared to GluA2-containing AMPARs^24^. Therefore, we compared EPSC amplitudes of transfected cells with neighbouring non-transfected control cells (Fig. 4d). Cortactin knockdown caused a significant increase in EPSC amplitude (control 141.4 ± 21.5 pA vs. transfected 182.9 ± 22.0 pA), which was rescued by expression of sh-resistant WT-cortactin (control 129.3 ± 35.5 pA vs transfected 132.5 ± 28.3 pA), but not by sh-resistant ΔR156-cortactin (control 83.2 ± 28.4 pA vs transfected 121.8 ± 40.1 pA), supporting our conclusion that disruption of GluA2-cortactin interactions causes the synaptic expression of GluA2-lacking AMPARs.

Taken together, these results demonstrate that the interaction between cortactin and GluA2 is required for maintaining surface and synaptic levels of GluA2-containing AMPARs under basal conditions.

### Disrupting GluA2-cortactin interaction causes lysosomal targeting and degradation of GluA2-containing AMPARs

**Figure 5:**
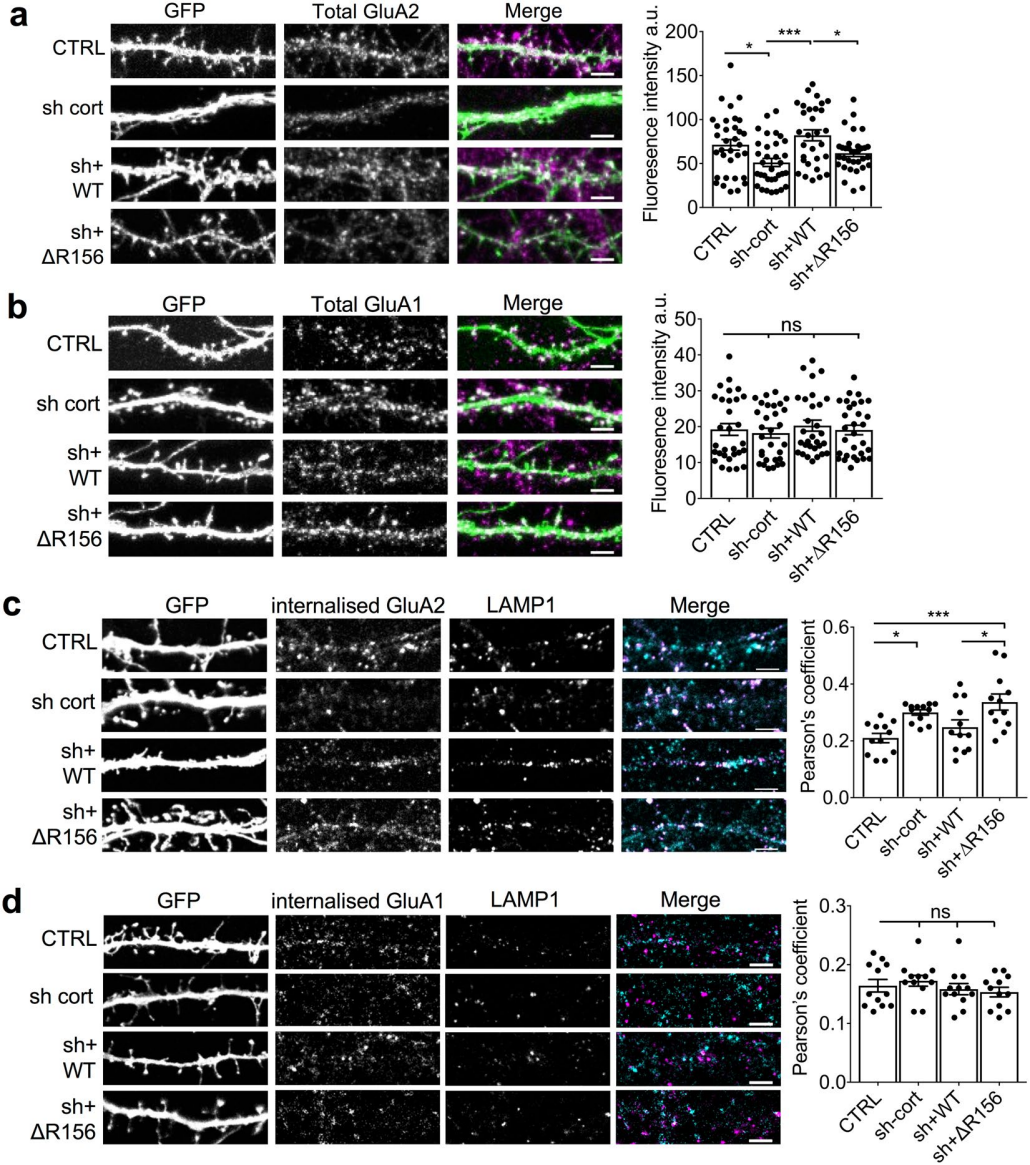
ΔR156-cortactin expression causes an increase in lysosomal targeting of GluA2 and reduced levels of total GluA2. (**a**) ΔR156-cortactin reduces total levels of endogenous GluA2. Hippocampal neurons transfected with GFP, cortactin shRNA + GFP, cortactin shRNA + sh-resistant GFP-WT-cortactin or cortactin shRNA + sh-resistant GFP-ΔR156-cortactin were permeabilized and stained with GluA2 antibody. Representative 45 μm lengths of dendrites are shown, scale bar = 5 µm. Graph shows fluorescence intensity for total GluA2 staining, values represent mean ± SEM. One-way ANOVA with Bonferroni’s test for multiple comparisons (F(3,125) = 4.48, *p < 0.05, ***p < 0.001, n = 30–33). (**b**) ΔR156-cortactin has no effect on total levels of endogenous GluA1. Hippocampal neurons transfected with constructs as in A, were permeabilized and stained with GluA1 antibody to label surface and internal receptors. Representative 45 μm lengths of dendrites are shown, scale bar = 5 µm. Graph shows fluorescence intensity for total GluA1 staining, values represent mean ± SEM. One-way ANOVA with Bonferroni’s test for multiple comparisons (F(3,116) = 0.3551, n = 30). (**c**) ΔR156-cortactin increases lysosomal targeting of GluA2. GluA2 antibody was applied to live hippocampal neurons transfected with constructs as in A, to label surface receptors. After washing, cultures were returned to the incubator for 60 min prior to fixation and permeabilization. Internalized GluA2 was labelled with AlexaFluor647**-**conjugated secondary antibody (cyan), and lysosomes were identified by staining with LAMP1 (magenta). Representative 45 μm lengths of dendrites are shown, scale bar = 5 µm. Graph shows Pearson’s colocalization coefficients for internalized GluA2 and LAMP1, values represent mean ± SEM. One-way ANOVA with Bonferroni’s test for multiple comparisons (F(3,44) = 3.254, *p < 0.05, ***p < 0.001, n = 12). (**d**) ΔR156-cortactin has no effect on lysosomal targeting of GluA1. Experiment was the same as C, above, except GluA1 antibody was used instead of GluA2. Representative 45 μm lengths of dendrites are shown, scale bar = 5 µm. Graph shows Pearson’s colocalization coefficients for internalized GluA1 and LAMP1, values represent mean ± SEM. One-way ANOVA with Bonferroni’s test for multiple comparisons (F(3,44) = 0.7693, n = 12).

To investigate whether the observed changes in surface GluA2 were accompanied by changes in total subunit expression level, we carried out immunocytochemistry on detergent-permeabilized neurons. Showing a similar pattern as the surface staining, total GluA2 was significantly reduced by expression of cortactin shRNA, which was fully rescued by sh-resistant WT-cortactin but not by sh-resistant ΔR156-cortactin (Fig. 5a). In contrast, total expression of GluA1 was unaffected by manipulation of cortactin expression (Fig. 5b).

The reduction in total GluA2 suggested that loss of cortactin function might have caused lysosomal degradation of GluA2-containing AMPARs. To test this hypothesis, we analyzed the constitutive trafficking of internalized GluA2 to LAMP1-positive lysosomes following immunolabelling of surface GluA2 in live neurons (“antibody feeding”). Using this technique, only AMPARs that originated on the cell surface and therefore trafficked through the endosomal system are labelled. Figure 5c shows that cortactin knockdown by shRNA caused a significant increase in the colocalization of internalized GluA2 with LAMP1, which was rescued by co-expression of sh-resistant WT-cortactin, but not sh-resistant ΔR156-cortactin. This demonstrated that cortactin is required for targeting GluA2-containing AMPARs away from lysosomes by direct interaction with GluA2 subunit. In contrast, GluA1 trafficking to LAMP1-positive lysosomes was unaffected by cortactin knockdown or by ΔR156-cortactin expression (Fig. 5d). Taken together with the data presented in Fig. 3, these results suggest a mechanism whereby cortactin directs GluA2-containing AMPARs to a recycling pathway. Loss of the interaction between cortactin and GluA2 causes a significant proportion of GluA2-containing AMPARs to be targeted to lysosomes for degradation, resulting in a reduction in synaptic GluA2 content.

**Figure 6:**
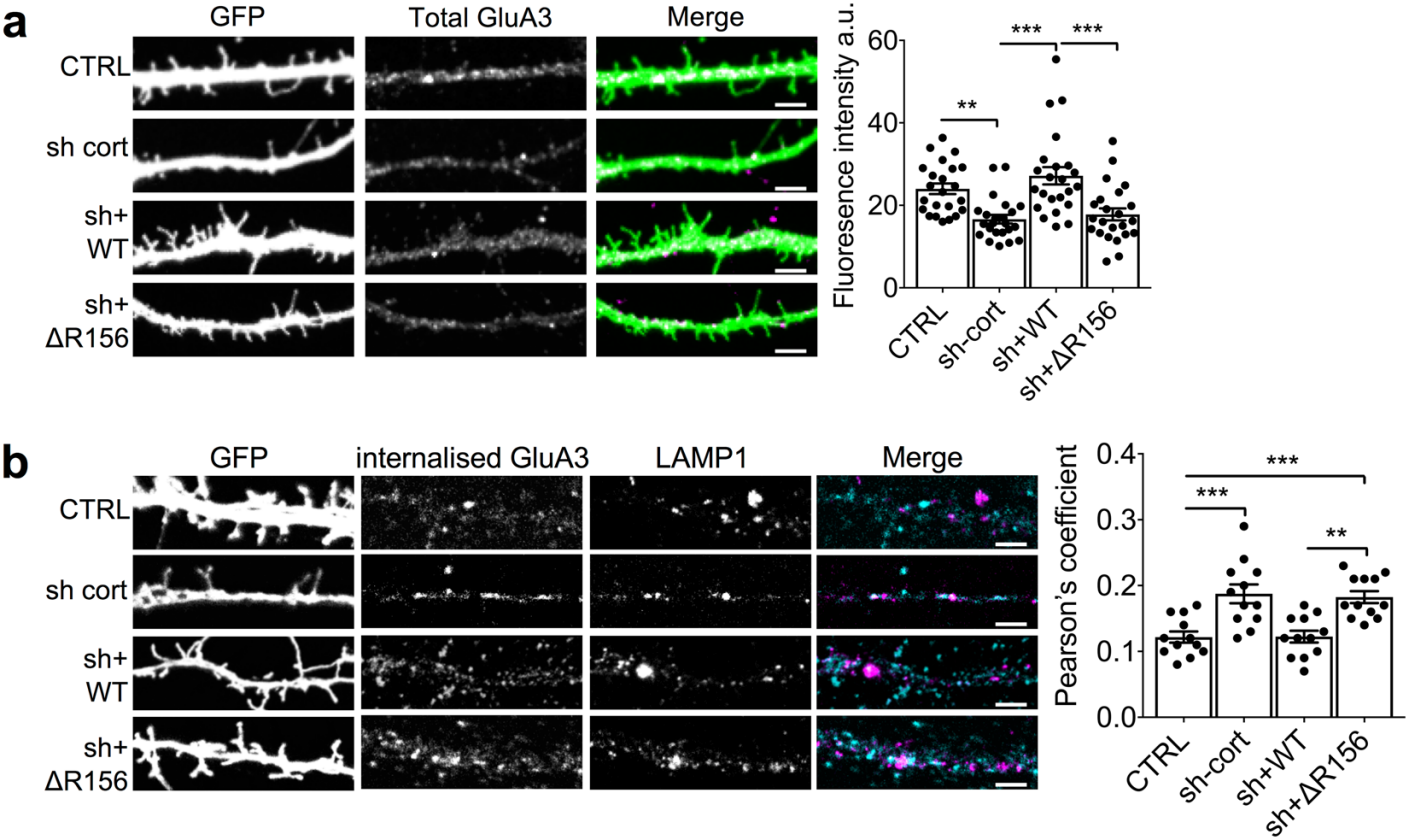
ΔR156-cortactin expression causes an increase in lysosomal targeting of GluA3 and reduced levels of total GluA3. (**a**) ΔR156-cortactin reduces total levels of endogenous GluA3. Hippocampal neurons transfected with GFP, cortactin shRNA + GFP, cortactin shRNA + sh-resistant GFP-WT-cortactin or cortactin shRNA + sh-resistant GFP-ΔR156-cortactin were permeabilized and stained with GluA3 antibody. Representative 45 μm lengths of dendrites are shown, scale bar = 5 µm. Graph shows fluorescence intensity for total GluA3 staining, values represent mean ± SEM. One-way ANOVA with Bonferroni’s test for multiple comparisons (F(3,66) = 11.94, ***p < 0.0001, n = 23). (**b**) ΔR156-cortactin increases lysosomal targeting of GluA3. GluA3 antibody was applied to live hippocampal neurons transfected with constructs as in a, to label surface receptors. After washing, cultures were returned to the incubator for 60 min prior to fixation and permeabilization. Internalized GluA3 was labelled with AlexaFluor647**-**conjugated secondary antibody (cyan), and lysosomes were identified by staining with LAMP1 (magenta). Representative 45 μm lengths of dendrites are shown, scale bar = 5 µm. Graph shows Pearson’s colocalization coefficients for internalized GluA3 and LAMP1, values represent mean ± SEM. One-way ANOVA with Bonferroni’s test for multiple comparisons (F(3,44) = 12.08, ***p < 0.0001, n = 12).

AMPARs on pyramidal neurons are composed predominantly of GluA1/A2 and GluA2/A3 heteromeric complexes, with GluA2 homomers thought to be rare or non-existent^25,26^. Since our results indicate that GluA1-containing receptors are not regulated by cortactin, we therefore hypothesised that the pool of GluA2 that is regulated by this mechanism is in complex with GluA3, and analysed total levels of GluA3 in neurons by immunocytochemistry. Showing a similar pattern as GluA2, total GluA3 was significantly reduced by expression of cortactin shRNA, which was fully rescued by sh-resistant WT-cortactin but not by sh-resistant ΔR156-cortactin (Fig. 6a). In further support of our hypothesis, cortactin knockdown by shRNA caused a significant increase in the trafficking of internalized GluA3 to LAMP1-positive lysosomes, which was rescued by co-expression of sh-resistant WT-cortactin, but not by sh-resistant ΔR156-cortactin (Fig. 6b). These results strongly suggest that cortactin preferentially regulates the trafficking of AMPARs containing GluA2/A3.

### Hippocampal CA1 LTD involves a pool of GluA2-containing AMPARs regulated by cortactin

**Figure 7:**
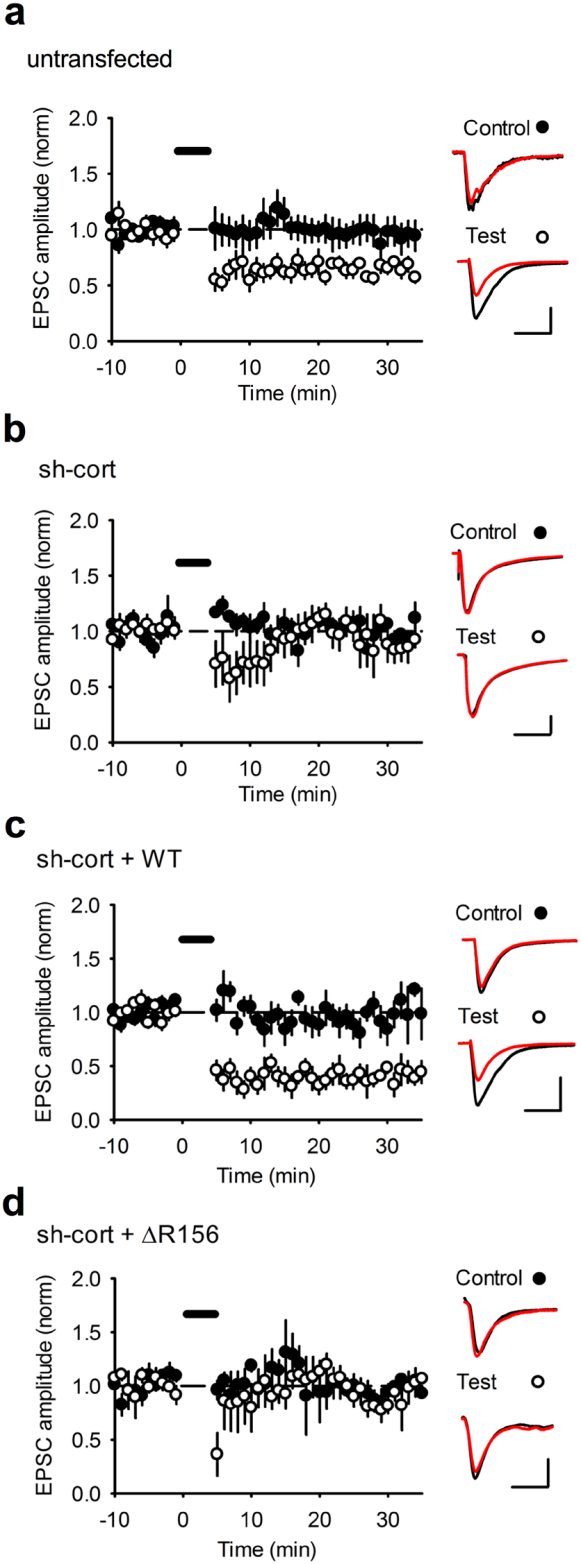
ΔR156-cortactin expression occludes hippocampal LTD. Organotypic hippocampal slice cultures were untransfected (**a**) or biolistically transfected with plasmids as described below (**b**–**d**). Values are mean ± SEM of EPSC amplitude normalized to baseline (closed circles = control input; filled circles = test input). (**a**) Stable AMPAR-mediated EPSCs and input-specific NMDAR-dependent LTD was recorded in control (nontransfected) cells (n = 7). (**b**) Input-specific LTD was completely abolished in cells transfected with cortactin shRNA (n = 5). (**c**) Input-specific LTD was consistently observed in cells transfected with cortactin shRNA plus GFP-WT-cortactin (n = 5). (**d**) Input-specific LTD was completely abolished in cells transfected with cortactin shRNA plus GFP- ΔR156-cortactin (n = 5).

Since LTD involves trafficking of AMPARs to lysosomes and their subsequent degradation^9,10^, and our results thus far suggest a role for cortactin in restricting lysosomal targeting of GluA2 and consequently controlling surface and synaptic AMPARs, we hypothesized that the cortactin-GluA2 interaction might be involved in LTD expression. To test this, we carried out electrophysiological recordings from CA1 pyramidal neurons in organotypic slices, using a low-frequency stimulation pairing protocol to induce LTD. Reliable LTD of AMPAR EPSCs could be induced in non-transfected control neurons (Fig. 7a; control 95.8 ± 11.9% vs test 65.2 ± 3.2%, n = 7, *p* < 0.05). In contrast, LTD was completely absent in neurons expressing cortactin shRNA (Fig. 7b; control 96.4 ± 15.5% vs test 104.1 ± 1.0%). LTD was rescued by co-expression of sh-resistant WT-cortactin (Fig. 7c; control 94.5 ± 10.7% vs test 40.7 ± 6.6%, n = 5, *p* < 0.01), but not ΔR156-cortactin and (Fig. 7d; control 105.0 ± 11.4% vs test 106.3 ± 7.1%, n = 5). Since ΔR156-cortactin caused a loss of surface and synaptic GluA2 under basal conditions, the absence of plasticity represents an occlusion rather than a block of LTD. Therefore, these data suggest that LTD involves a disruption of GluA2-cortactin interactions to target GluA2-containing AMPARs to lysosomes.

### cLTD induction causes a reduction in GluA2-cortactin interaction via Src-mediated phosphorylation

**Figure 8:**
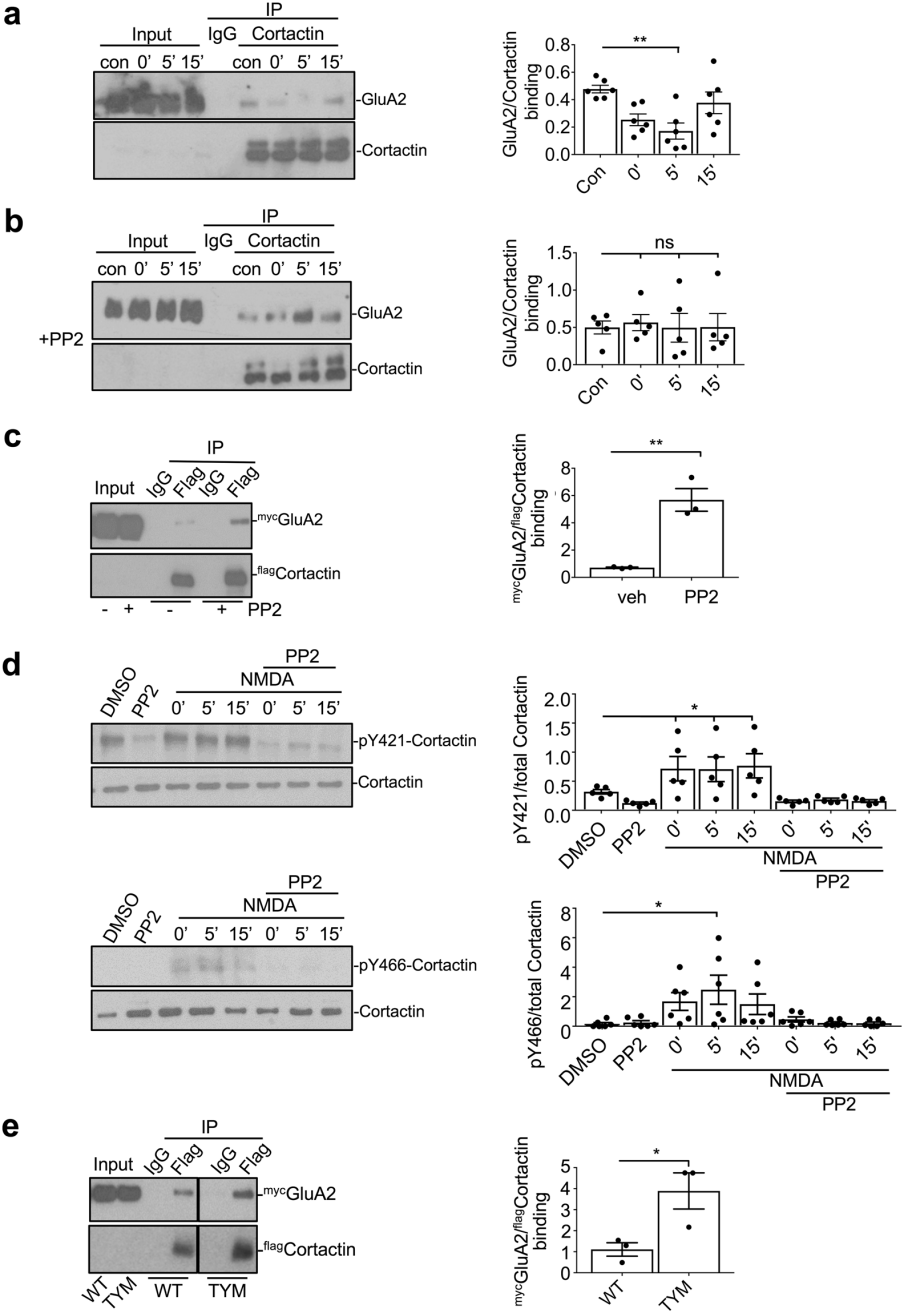
cLTD causes transient dissociation of GluA2 from cortactin via Src-mediated phosphorylation. (**a**) cLTD causes a rapid and transient dissociation of GluA2 from cortactin. Neurons were exposed to NMDA for 3 min to induce cLTD. Lysates were prepared at 0, 5 and 15 min after the end of stimulation and immunoprecipitated with cortactin antibody or IgG control. Bound proteins were detected by Western blotting. Graph shows quantification of GluA2 bound to cortactin, values represent mean ± SEM. One-way ANOVA with Bonferroni’s test for multiple comparisons (F(3,20) = 1.927, **p < 0.01, n = 6). (**b**) Src-family kinases are required for cLTD-induced dissociation of GluA2 from cortactin. Neurons and lysates were treated as in A, except the Src inhibitor PP2 was added 30 min prior to cLTD induction. Graph shows quantification of GluA2 bound to cortactin, values represent mean ± SEM. One-way ANOVA with Bonferroni’s test for multiple comparisons (F(3,16) = 0.5583, n = 5). (**c**) Src-family kinase activity regulates GluA2-cortactin interaction in heterologous cells. HEK293 cells expressing ^myc^GluA2 and ^flag^WT-cortactin were treated with 10 µM PP2 or vehicle (DMSO) for 30 min prior to cell lysis. Lysates were immunoprecipitated with flag antibody and bound proteins were detected by Western blotting. Graph shows quantification of ^myc^GluA2 bound to ^flag^cortactin, values represent mean ± SEM. Unpaired two-tailed student’s t-test (t(4) = 5.995, **p < 0.01, n = 3). (**d**) cLTD induction causes an increase in cortactin phosphorylation at Y421 and Y466. Neurons were exposed to NMDA for 3 min to induce cLTD, in the absence or presence of the Src inhibitor PP2 as shown. Lysates were prepared at 0, 5 and 15 min after the end of stimulation. Phosphorylation at Y421 and Y466 was analysed by Western blotting using total-cortactin and cortactin phospho-specific antibodies. Graphs show quantification of phospho/total cortactin, values represent mean ± SEM. Y421, one-way ANOVA with Bonferroni’s test for multiple comparisons (F(7,28) = 6.298, *p < 0.05, n = 5). Y466, one-way ANOVA with Bonferroni’s test for multiple comparisons (F(7,35) = 4.366, *p < 0.05, n = 6). (**e**) Cortactin phospho-null mutations enhance GluA2 binding. Lysates were prepared from HEK293 cells expressing ^myc^GluA2 and ^flag^WT-cortactin or ^flag^TyrM-cortactin, and immunoprecipitated with anti-flag antibody. Bound proteins were detected by Western blotting. Graph shows quantification of ^myc^GluA2 bound to ^flag^cortactin, values represent mean ± SEM. Unpaired two-tailed student’s t-test (t(4) = 3.031, *p < 0.05, n = 3).

To directly test the hypothesis that GluA2-cortactin interactions are disrupted in response to LTD induction, we performed co-IPs to isolate endogenous protein complexes from cultured neurons, and used a chemical LTD (cLTD) protocol in which NMDARs are activated by bath application of NMDA. Consistent with our hypothesis, cLTD caused a significant reduction in the interaction between GluA2 and cortactin at 5 min after NMDAR stimulation (Fig. 8a). Interestingly, this reduction was transient, with the interaction returning to baseline levels by 15 min after stimulation. Cortactin is known to be regulated by phosphorylation of a number of tyrosine residues towards the C-terminus, in particular Y421, Y466 and Y482, which regulate its interaction with protein binding partners^27^. Moreover, our GST pull-down assays demonstrated that a cortactin truncation mutant lacking the C-terminal half of the protein bound more strongly to GluA2 (Fig. 1f), suggesting that this region of cortactin negatively regulates the interaction with GluA2. We therefore asked whether phosphorylation of cortactin C-terminal tyrosine residues regulates binding to GluA2 in response to cLTD induction. Since Src family kinases (SFKs) are thought to mediate the majority of phosphorylation events in the cortactin C-terminus^27^, we investigated whether inhibition of SFKs affected the cLTD-induced reduction in GluA2-cortactin binding. In agreement with this hypothesis, application of the SFK inhibitor PP2 to neuronal cultures during cLTD induction completely blocked the effect of NMDA (Fig. 8b). To investigate directly the effect of SFK activity on GluA2-cortactin interactions, we carried out co-IPs from HEK cells expressing ^myc^GluA2 and ^flag^cortactin. Application of PP2 for 30 min prior to cell lysis caused a dramatic increase in GluA2-cortactin binding compared to controls (Fig. 8c), strongly suggesting that SFK activity disrupts this interaction.

To investigate which cortactin tyrosine residues are phosphorylated in response to cLTD induction, we used Western blotting with previously-characterized phospho-specific antibodies against cortactin phosphorylated at Y421 or Y466^28,29^. Phosphorylation of both Y421 and Y466 was increased by cLTD induction, which was blocked in both cases by PP2 (Fig. 8d). We were unable to detect signal with a Y482 antibody. To test directly the role of these tyrosine residues in regulating GluA2 binding, we carried out co-IPs from HEK293 cells transfected with ^flag^WT-cortactin or a mutant with Y421, Y466 and Y482 mutated to phenylalanine to block phosphorylation^30^, and analysed binding to ^myc^GluA2. The tyrosine mutant (^flag^TYM-cortactin) showed significantly greater binding to GluA2 compared to wild-type (Fig. 8e), consistent with the hypothesis that tyrosine phosphorylation inhibits the interaction. Taken together, these results demonstrate that cortactin dissociates from GluA2 in response to cLTD induction via a mechanism that involves phosphorylation of cortactin C-terminal tyrosine residues by SFKs.

## Discussion

We define a new role for cortactin as a regulator of AMPAR endosomal sorting by direct interaction with GluA2 subunit. GluA2 has three independent binding sites in repeats 1, 5 and 6 of the cortactin repeat region. Mutating all three GluA2 binding sites on cortactin causes the targeting of GluA2/GluA3-containing receptors to lysosomes and their consequent degradation, resulting in a loss of surface and synaptic GluA2 under basal conditions. This loss of GluA2 at the synapse caused by disrupting its binding to cortactin occludes subsequent LTD expression. Furthermore, we show that cLTD induction causes a dissociation of endogenous cortactin from GluA2 via phosphorylation of cortactin on residues Y421 and Y466. These results demonstrate that cortactin functions to maintain GluA2/A3 levels by directing receptors away from lysosomal degradation, and suggest that disrupting GluA2-cortactin interactions to target GluA2/A3 to lysosomes is an essential component of LTD expression.

We used a molecular replacement approach, in which endogenous cortactin expression was knocked down by shRNA, and replaced by GFP-tagged WT or mutant cortactin. While the recombinant proteins were expressed at a significantly higher level than endogenous cortactin, the WT rescue construct rescued F-actin, spine density, synapse density, AMPAR trafficking and synaptic function to levels that were indistinguishable from controls. This indicated that there were no detrimental effects of cortactin overexpression *per se*.

### Cortactin as a regulator of endo-lysosomal trafficking

The targeting of AMPARs to lysosomes instead of recycling to the cell surface has been shown previously to be an essential step in reducing synaptic AMPARs during LTD expression^9–11^. Our results define a role for cortactin in endosomal sorting to lysosomal compartments. Interestingly, a role for the region of GluA2 that we define here as binding cortactin was shown before to be a critical determinant in restricting GluA2 from trafficking to lysosomes^9^. Lee *et al*. showed that deleting residues 849–853 in GluA2 C-terminus diverted recombinant GluA2 to lysosomes instead of recycling endosomes^9^. The Δ849–853 mutation has been used in many studies to disrupt GluA2 binding to NSF, and therefore NSF was suggested to be the major determinant of recycling. Our results suggest an alternative interpretation; that cortactin binding to the same region of GluA2 maintains a recycling pool of GluA2 away from lysosomal degradation.

While cortactin has been defined in a trafficking context predominantly as an endocytic protein^17,31^, previous reports have also suggested a role for cortactin in endo/lysosomal sorting in non-neuronal cells^18^. Cortactin was observed localized to tubular domains on sorting endosomes, which are thought to be the sites where specific cargo proteins are clustered in a first step towards recycling to the plasma membrane. Cortactin was shown to be required for the localization of the β2-adrenergic receptor (β2AR) in recycling tubules on the endosome^18^. This mechanism for β2AR recycling appears to bear similarities to the mechanism we define here for GluA2-containing AMPARs. However, there are also notable differences. Interestingly, it has been shown that β2AR recycling requires cortactin phosphorylation at Y466 by Src^32^, whereas our results suggest that Src phosphorylation disrupts GluA2-cortactin, which would favour GluA2 degradation. We show that GluA2 binds directly to three independent sites on cortactin, and disrupting these interactions causes GluA2 to traffic to lysosomes. In contrast, cortactin does not, to our knowledge, bind β2AR. Our results suggest that a single cortactin molecule has the potential to bind three GluA2 subunits, which might be important for clustering AMPARs in endosomal subdomains in preparation for recycling to the cell surface. Future work will unravel the detailed molecular mechanisms that underlie cortactin-dependent GluA2 trafficking.

Cortactin phosphorylation by Src at Y421 and Y466 has been shown previously to modulate cortactin protein-protein interactions in non-neuronal cells^27,33^. In invadopodia, the regulation of actin polymerization by cortactin is modulated by phosphorylation at these sites by controlling interactions with regulatory components of the actin cytoskeleton^34^. It has also been suggested that cortactin binding to dynamin-2 is enhanced by Src-mediated tyrosine phosphorylation of cortactin, which regulates transferrin receptor endocytosis^35^. Our results indicate that phosphorylation at Y421 and Y466 by Src inhibits GluA2 binding in response to the induction of cLTD. Since GluA2 binds cortactin in the repeat region (residues 85–336), it is likely that Y421 and Y466 affect GluA2 binding by affecting intramolecular interactions. This is supported by our observation that deleting the C-terminal half of cortactin, which includes Y421 and Y466, dramatically enhanced binding to GluA2 *in vitro*.

Cortactin promotes Arp2/3-dependent actin polymerization, and this aspect of its function is proposed to be involved in the endosomal recycling of β2AR^15,18^. PICK1 also associates with GluA2, and regulates both endocytosis and recycling of GluA2-containing AMPARs^36,37^. We previously demonstrated that PICK1 inhibits Arp2/3 activity^38^ (but see^39^), and that PICK1-mediated Arp2/3 inhibition is required for LTD^40,41^. Taken together, these studies suggest that GluA2 can associate with both an Arp2/3 activator and an Arp2/3 inhibitor, leading to the intriguing possibility that PICK1 and cortactin act as antagonistic regulators of actin dynamics at endosomes to bidirectionally control AMPAR recycling.

### Subunit-specificity

While it is not surprising that a GluA2 C-tail interacting protein shows specificity for GluA2 over GluA1 in a reduced system, our observation that cortactin loss-of-function in neurons preferentially affects GluA2/A3-containing AMPARs but not GluA1-containing AMPARs was unexpected, and suggests a mechanism for preventing GluA1/A2 complexes from interacting with cortactin. Since we are not aware of any precedent for GluA1 or GluA3 influencing GluA2 binding to accessory proteins, a biochemical explanation for this observation seems unlikely. Perhaps this specificity could be explained by subcellular localization of distinct AMPAR subtypes; other as yet undefined GluA1-dependent mechanisms might restrict GluA1-containing receptors from entering the specific endosomal domain regulated by cortactin.

Previously-described mechanisms for regulating endo-lysosomal sorting of AMPARs vary in their subunit-specificity and upstream signalling requirements. It has been shown that the AMPAR auxiliary protein stargazin associates with the adaptor protein complex AP3, which is required for late endosomal targeting of GluA1 and GluA2-containing AMPARs and LTD^11^. Ubiquitination of GluA1 by Nedd4-1 targets GluA1 subunit to lysosomes in response to stimulation with AMPA^42^. Importantly, NMDAR stimulation does not affect Nedd4-1 dependent ubiquitination of GluA1, suggesting that this mechanism is not involved in LTD. GluA2 subunit is not ubiquitinated by Nedd4-1, although it is unclear whether GluA1/2 heteromeric receptors are affected. However, more recent reports have shown that GluA2 is ubiquitinated, which targets both GluA1 and GluA2 to lysosomes in response to stimulation with AMPA, but not NMDA^43,44^. Hence the cortactin-dependent sorting we describe here differs from previous reports in being a subunit-specific mechanism that is involved in NMDAR-dependent synaptic plasticity.

A GluA2-dependent trafficking mechanism suggests the potential for regulating the subunit composition of synaptic AMPARs. Indeed, our electrophysiology data demonstrate that mutating the GluA2 binding sites on cortactin causes rectification of AMPAR EPSCs, which indicates the presence of GluA2-lacking Ca^2+^-permeable (CP-)AMPARs at synapses. Since we observe no change in surface GluA1 following the same manipulation, we propose that CP-AMPARs are recruited to synapses by lateral diffusion from extrasynaptic sites, replacing the GluA2/A3 receptors that are lost to lysosomal degradation. Our rectification data indicate that only a proportion of synaptic AMPARs are replaced by CP-AMPARs, but whether the degraded GluA2/A3 receptors are fully replaced by CP-AMPARs, or if there is a net change in AMPAR receptor number is unclear.

It has recently been shown that hippocampal LTD involves a transient recruitment of CP-AMPARs to synapses^45^. While this has been shown to depend on the activities of AKAP150-anchored PKA and calcineurin, additional components to this mechanism are likely to be revealed in the future. The rapid and transient reduction in GluA2-cortactin binding that we observed in response to cLTD induction could contribute to a mechanism for the short-term recruitment of CP-AMPARs to synapses. A number of forms of plasticity have emerged in the past decade that involve the transient synaptic recruitment of CP-AMPARs to mediate essential Ca^2+^ influx for various downstream signalling events that are needed for the maintenance of plasticity^46–48^. Furthermore, numerous disease states also involve a longer-term recruitment of CP-AMPARs to synapses, which can lead to neuronal dysfunction and/or cell death^49–51^. It will be interesting to determine whether the regulation of synaptic GluA2 content by cortactin is involved in such physiological or pathological forms of plasticity, and might therefore be a novel therapeutic target.

## Methods

### Plasmids and plasmid construction

His_6_-tagged proteins were expressed from pET28 (Novagen), GST-fusions from pGEX4T-1 (Pharmacia) in BL21 bacteria. For HEK293 cells, WT and ΔR156 cortactin were expressed from pcDNA3.1. TYM cortactin was a kind gift from Prof. S. Weed. Multiple sequence alignments were diagrammatized using Jalview Version 2. Molecular replacement experiments were performed using the modified pFUGW vector and was a kind gift from Prof. R. Malenka, as described previously^52^. Control constructs did not contain shRNA. The H1 RNA III pol promoter drove shRNA and EGFP tagged cortactin rescues were driven by the hUbC promoter. Cortactin shRNA targeted base pairs 300–319 of the rat and mouse sequence, as follows ‘5’-GCACTGCTCACAAGTGGAC-3′ and was previously validated^19^. Silent mutations were introduced to make sh-resistant cortactin: GCAtTGtTCtCAgGTGGAC (mutations in lower case).

### Preparation of recombinant proteins

His_6_ and GST fusions were expressed and purified as described previously (Hanley *et al*., 2002).

### Antibodies

The antibodies used were as follows: anti-cortactin (clone 4F11, Millipore); anti-GluA1 (Calbiochem); anti-GluA2 for Westerns (Synaptic systems); anti-GluA2 for co-IPs (MAB397, Millipore); anti-GluA2 for immunocytochemistry (BD Pharminogen, mouse); anti-GluA3 (Alomone, rabbit); anti-phosphoY421-cortactin (Sigma); anti-phosphoY466-cortactin (Millipore); anti-phosphoY482-cortactin (Millipore); anti-Myc (Santa Cruz); anti-Flag (Sigma); anti-GST (Cell Signalling Technologies); anti-GFP (Chromtek); anti-LAMP1 (Abcam); anti-PSD-95 (Millipore); control IgG (Thermo).

### Buffers

Lysis buffer: 25 mM HEPES, pH7.4, 150 mM NaCl, 1% Triton X-100, protease inhibitor cocktail (Roche), phosphatase inhibitor II, III (Sigma).

HTG buffer: 50 mM HEPES, pH7.5, 150 mM NaCl, 1% Trition X-100, 10% glycerol.

Harsh HTG buffer: 20 mM Tris pH7, 500 mM NaCl, 5 mM imidazole, 2 mM MgCl_2_, 1% Triton, 1 mM DTT. G-actin buffer: 5 mM Tris-HCl, pH 8.0, 0.2 mM CaCl2.

Actin polymerization buffer: 10 mM Tris, pH 7.5, 50 mM KCl, 2 mM MgCl_2_, 1 mM ATP.

HBS: 25 mM HEPES, pH7.5, 137 mM NaCl, 5 mM KCl, 15 mM glucose, 1.5 mM CaCl_2_, 0.8 mM MgCl_2_.

### Coimmunoprecipitations

Coimmunoprecipitations were performed as essentially described (Hanley and Henley, 2005). Cultures were lysed in lysis buffer. Lysates containing 150 µg total protein were incubated with 3 µg antibody for 3 h at 4 °C, followed by addition of protein G-sepharose (Sigma) for 1 h at 4 °C. Following extensive washing, bound proteins were detected by Western blotting.

### GST pulldown assays

5 µg GST or GST-GluA2C was immobilized on glutathione agarose beads in lysis buffer for 60 mins at 4 °C. Beads were washed in HTG buffer and then incubated with 25 nM His_6_ cortactin for a further 60 mins at 4 °C. Beads were then washed twice in harsh HTG buffer and once in normal HTG buffer before bound proteins were detected by Western blotting.

### Actin co-sedimentation assays

Desiccated rabbit muscle G-actin (Cytoskeleton) was reconstituted in actin buffer and left on ice for 30 min before diluting 10 × actin polymerization buffer to 1 ×. This F-actin stock was incubated at room temperature for 1 h to polymerise the actin filaments and kept at 4 °C until use for up to a week. Meanwhile, purified His_6_-WT/ΔR156 cortactin was ultracentrifuged at 55,000 rpm for 1 h at 4 °C and supernatants removed and placed on ice. 1.25 mM clarified his_6_ cortactin was incubated with 5 µM F-actin for 30 min at room temperature and then ultracentrifuged at 24 °C for 1.5 hr at 55000 rpm. Pellets and supernatants were analyzed by SDS-PAGE and Coomassie staining.

### Cell culture, transfection and cLTD

HEK293T cells (ATCC CRL-1573) were transfected using linear Polyethylenimine (PEI, 25 kDa) (Fisher Scientific) and proteins were solubilized in ice cold lysis buffer 24–48 h later. Hippocampal neurons were prepared from E18 Wistar rats. For molecular replacement studies, DIV9 neurons were transfected with Lipofectamine 2000 and used for experiments 7 days later. Where specified, neurons were given a cLTD stimulus as follows: 50 µM NMDA, 20 µM glycine or plain HBS for 3 min at 37 °C and returned to conditioned media for an indicated period of time.

### Electrophysiology

Organotypic slices were prepared from P8 Wistar rats using the interface method^53^. Transverse hippocampal slices (400 μm) were placed on Millicell culture plate inserts (Millipore) and maintained at 35 °C, 5% CO_2_ in MEM-based culture media containing 20% horse serum and (in mM): 30 HEPES, 16.25 glucose, 5 NaHCO_3_, 1 CaCl_2_, 2 MgSO_4_, 0.68 ascorbic acid, and 1 μg/ml insulin (pH 7.28 and 320 mOsm). Biolistic transfection was performed using a Helios GeneGun (Bio-Rad) and whole-cell voltage-clamp recordings were made from CA1 pyramidal cells (Vh = −70 mV) 4–7 days later. Recordings were made in ACSF containing the following (mM): 119 NaCl, 2.5 KCl, 1 NaH_2_PO_4_.H_2_O, 26.2 NaHCO_3_, 11 glucose, 1.3 MgSO_4_, 2.5 saturated with 95% O_2_, 5% CO2. Picrotoxin (50–100 μM) and 2-chloroadenosine (1–2 μM) were routinely added to ACSF and bath temperature was maintained at 32 °C. Intracellular solution contained (in mM): 117 CsMeSO_3_, 8 NaCl, 10 HEPES, 5 QX314, 4 ATP-Mg. 0.3 GTP-Na, 0.5 EGTA adjusted to pH 7.4 with CsOH and ∼290 mOsM. For rectification measurements, 100 μM spermine was added to the intracellular solution in order to prevent dilution of cytoplasmic polyamines and 50 μM D-AP5 was added to the ACSF. For rectification and amplitude measurements, individual fluorescent transfected CA1 pyramidal neurons were identified, followed by a neighbouring no transfected neuron. EPSC amplitudes were measured at a holding potential of −70 mV. EPSC rectification at holding potentials of −70 mV, 0 mV and +40 mV. For rectification analysis, a ratio of the I/V plot slopes between −70 mV and 0 mV and between 0 mV and +40 mV data was calculated to generate a rectification index (RI = Gradient_+40 mV_/Gradient_−70 mV_). EPSC rectification and amplitudes were compared between transduced and non-transduced cells in the same slice (paired Student’s *t*-test). For LTD experiments, individual transfected neurons were recorded in each slice. Two bipolar tungsten stimulating electrodes were placed in the *stratum-radiatum* and stimulated at 0.1 Hz for a stable baseline of 10 mins. The LTD induction protocol consisted of 300 stimuli at 1 Hz given to the test pathway whilst holding the cell at −40 mV. LTD was assessed at 30–35 minutes following the induction protocol by statistical comparison between control and test pathways (paired Students *t*-test).

### Immunocytochemistry

Live DIV16 hippocampal neurons were surface labelled with anti-GluA2 (BD Pharminogen, mouse, 30 µg/ml) for 20 mins at room temperature in HBS, followed by washing in HBS. For surface staining experiments, neurons were then fixed in 4% paraformaldehyde (PFA) for 15 min and stained with anti-mouse AlexaFluor647. For antibody feeding experiments, cells were washed in HBS and returned to conditioned media for 60 min. Neurons were then fixed in PFA for 5 min and stained with anti-mouse AlexaFluor405 secondary. Following another 5 min fixation in PFA, cells were permeabilized and labelled with anti-LAMP1 (rabbit) antibody followed by anti-mouse AlexaFluor647 and anti-rabbit AlexaFluor563 secondary. For total staining experiments, neurons were fixed in PFA, permeabilized and stained with appropriate antibodies or AlexaFluor647 conjugated phalloidin (Invitrogen). Coverslips were mounted in Fluoromount (Electron Microscopy Sciences) and images were acquired on a SP5-AOBS (Leica) confocal laser scanning microscope attached to a DM I6000 (Leica) inverted epifluorescence microscope, using a 63 × oil immersion objective lens. Images were acquired with a resolution of 1024 × 1024 pixels at 1 × optical zoom, a pinhole of 1 airy unit (AU) at 400 Hz averaged 4 times and analyzed using FiJi software. For spine density analysis, each image consisted of a 2–2.5 μm z-stack at 0.2 μm intervals and maximum projections were taken for each series and analyzed with NeuronStudio^54^.

### Experimental Design and Statistical Analysis

All data are presented as mean ± SEM unless otherwise stated. Data were first tested for normality using the D’Agonstino-Pearson omnibus K2 normality test to determine the appropriate statistical test. Since all data with large sample size were found to fit a normal distribution, parametric statistics were employed. For experiments with n numbers lower than 8, data was assumed to fit a normal distribution without formal testing. Unpaired two-tailed Student’s t tests were used for statistical comparisons between 2 groups, and for experiments containing 3 or more groups, one-way analysis of variance (ANOVA) was employed. To compare all variables with one another, a Bonferroni’s post-hoc test for multiple comparisons was used. To compare each variable to a single control variable, a Dunnett’s post hoc test was used. All statistical tests were performed on GraphPad Prism (v6.0) and differences were considered significant if p < 0.05. *p < 0.05; **p <0.001; ***p < 0.0001.

### Data availability

The datasets generated during and/or analysed during the current study are available from the corresponding author on reasonable request.

## Electronic supplementary material

Supplementary information
