# Supplementary material for: Cortactin regulates endo-lysosomal sorting of AMPARs via direct interaction with GluA2 subunit
Source: Sci Rep. 2018 Mar 7;8:4155. doi: 10.1038/s41598-018-22542-z (PMC5841360; doi:10.1038/s41598-018-22542-z)

Supplementary information for:

Cortactin regulates endo-lysosomal sorting of AMPARs via direct interaction with GluA2 subunit.

Gabrielle T. Parkinson, Sophie E.L. Chamberlain, Nadia Jaafari, Matthew Turvey, Jack R. Mellor and Jonathan G. Hanley

1. Centre for Synaptic Plasticity and School of Biochemistry,

2. Centre for Synaptic Plasticity and School of Physiology, Pharmacology & Neuroscience

Biomedical Sciences Building,

University of Bristol,

University Walk,

Bristol BS8 1TD,

UK

*Correspondence

e-mail: jon.hanley@bristol.ac.uk

tel: +44 (0)117 3311944

Supplementary figure S1: Full-size blots/gels for all figures

Figure 1A

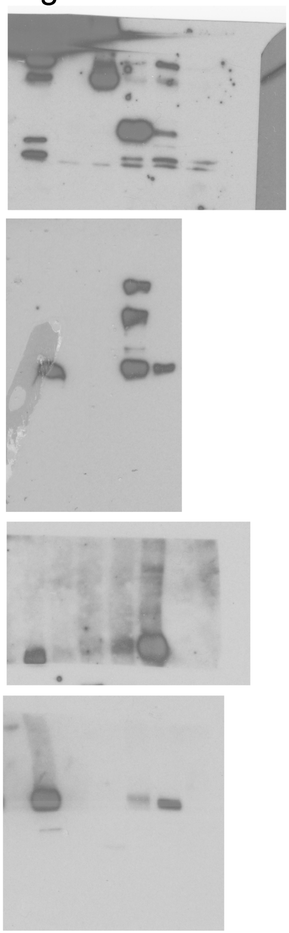

Figure 1B

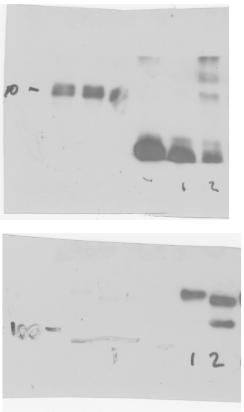

Figure 1C

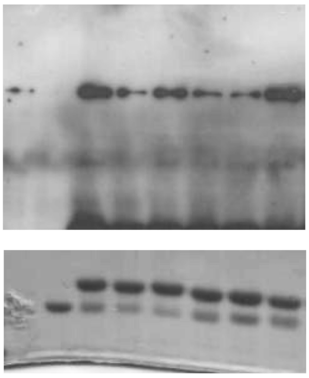

Figure 1E

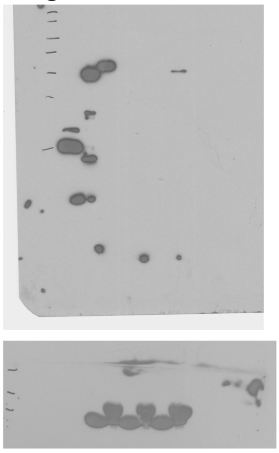

Figure 1F

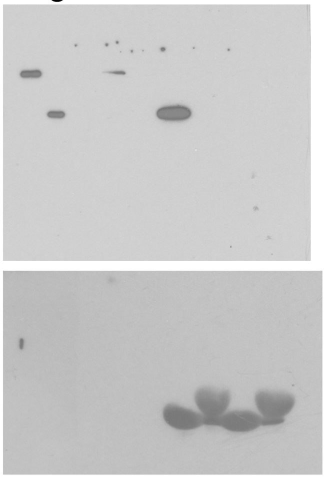

Figure 1G

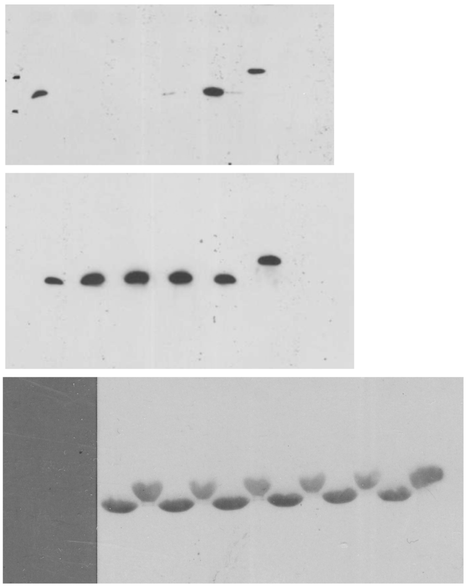

Figure 2C

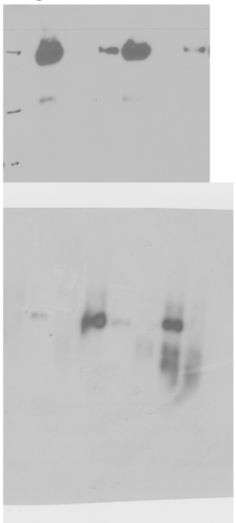

Figure 2B

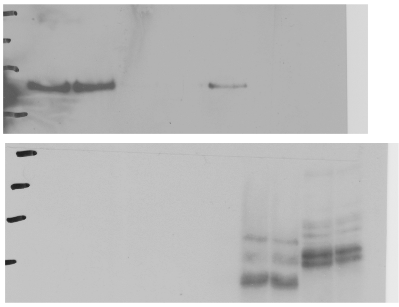

Figure 3A

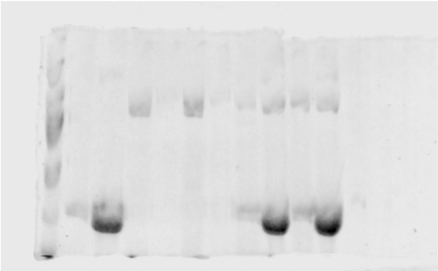

Figure 8A

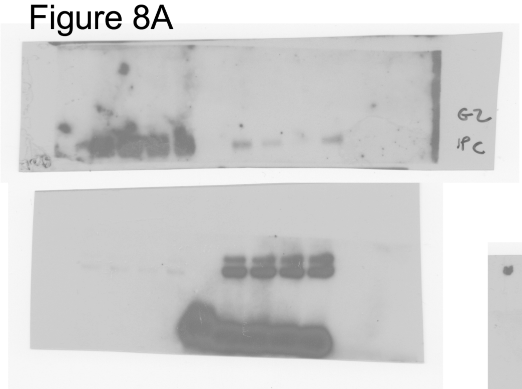

Figure 8B

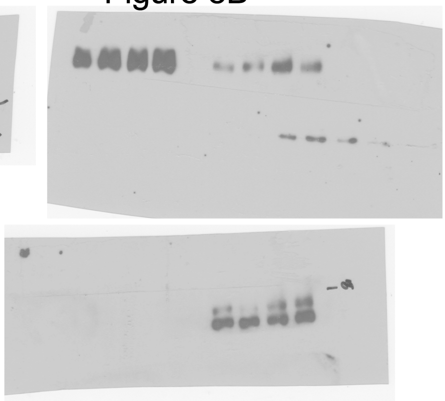

Figure 8C

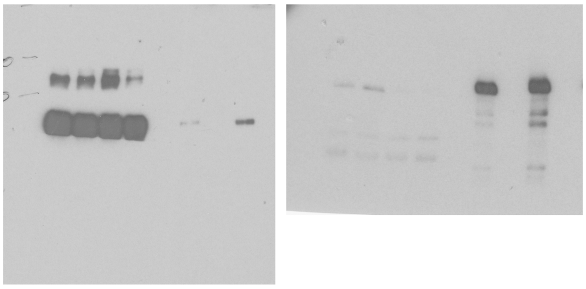

Figure 8E

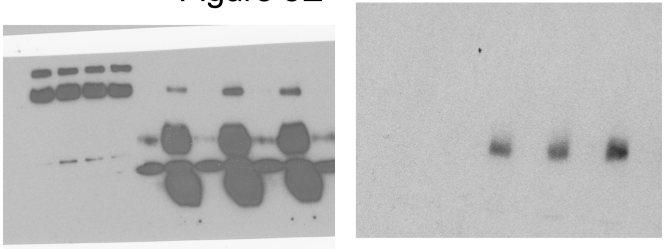

Supplement: Supplementary file 1 — Supplementary information [file 41598_2018_22542_MOESM1_ESM.pdf]
